# Supplementary material for: Metagenome-guided culturomics for the targeted enrichment of gut microbes
Source: Nat Commun. 2025 Jan 14;16:663. doi: 10.1038/s41467-024-55668-y (PMC11733127; doi:10.1038/s41467-024-55668-y)
Supplement: Supplementary file 1 — Supplementary Information [file 41467_2024_55668_MOESM1_ESM.pdf]

Title: Metagenome-guided culturomics for the targeted enrichment of gut microbes.

Jeremy Armetta, Simone S. Li, Troels Holger Vaaben, Ruben Vazquez-Uribe and Morten O. A. Sommer

Supplementary material

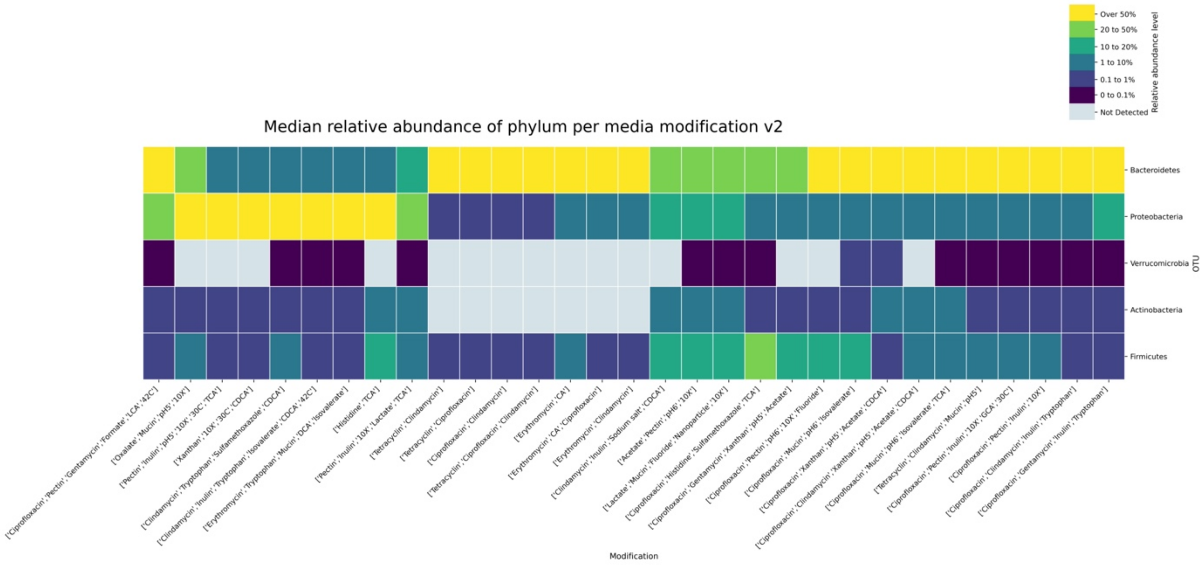

Supplementary Figure 1 - Median relative abundance of phylum per media modification

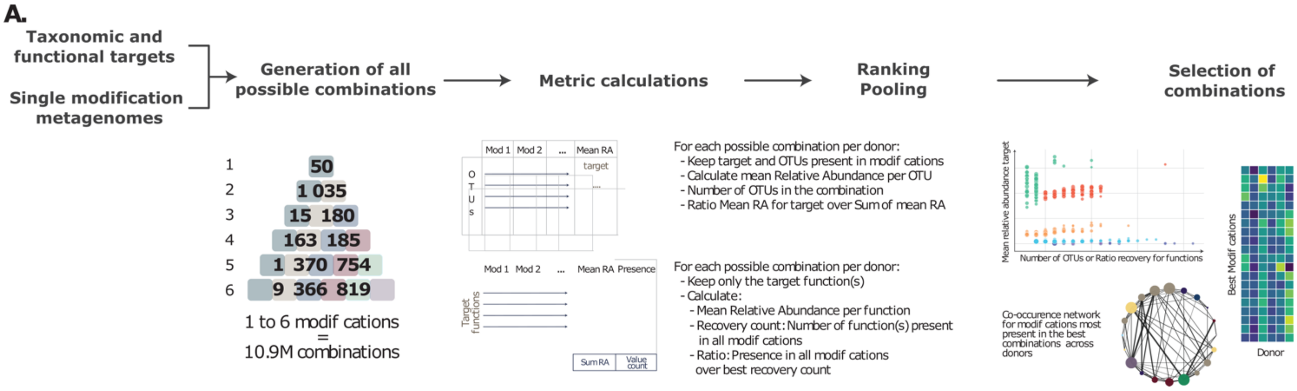

Supplementary Figure 2 – Design steps used to determine the components of media combinations.

Supplementary Table 1: List of media used in the study

This table describe the selective agent used in the different media variants during the Benchmark and enrichment phases of the study. Each variant is based on GAM-STD and contains a single selective agent. Enrichment media (ISO-XXXX) are composed of selective agents from Benchmark at the same final concentration.

Selective agent	Medium ID	Final agent concentration in medium	Notes
Standard - No selective agent	GAM-STD	NA	GAM without any selective agent, incubated anaerobically, used for reference.
**Antibiotics**			
Cefotaxime	GAM-CTX	16 mg/l	Broad spectrum, third gen cephalosporine in the B-lactam category with bactericidal action. Filter sterilized
Piperacillin	GAM-PIP	32 mg/l	Broad spectrum B-lactam penicillin with bactericidal action. Filter sterilized
Tetracycline	GAM-TET	32 mg/l	Protein synthesis inhibition. Filter sterilized
Erythromycin	GAM-ERY	32 µg/ml	Macrolide, broad spectrum against translocation in peptide synthesis. Filter sterilized
Clindamycin	GAM-CLIN	16 mg/l	E coli is efficiently removed with lincosamide while preserving Bacteroides and eubacterium/clostridium. Filter sterilized
Ciprofloxacin	GAM-CIP	16 mg/l	Fluoroquinolone, inhibit replication, broad spectrum, efficient to remove Enterobacteriaceae. Filter sterilized
Gentamycin	GAM-GEN	32 mg/l	Aminoglycoside, inhibit initiation, broad spectrum, inactive against most anaerobes. Filter sterilized

Imipenem	GAM-IMIPM	8 mg/l	Broad spectrum, carbapenem in the B-lactam category with bactericidal action. Filter sterilized
Niclosamide	GAM-NICLO	0.5 mg/l	Antiparasitic, potential for antibiotic and antiviral properties. Filter sterilized
Sulfamethoxazole	GAM-SLMET	64 mg/l	Against nucleic acid through purine synthesis inhibition, sulfonamide, broad spectrum. Inactive against anaerobes, efficient to remove Enterobacteriaceae. Filter sterilized
Chloramphenicol	GAM-CHLP	32 mg/l	Inhibit peptidyl transferase. Broad spectrum, useful to select for yeast. Filter sterilized
**Acids**			
Acetate	GAM-ACTAT	3 g/l	Acid form used to prepare the stock solution. pH adjusted to 7 with NaOH and filter sterilized
Butyrate	GAM-BUTY	2 g/l	Acid form used to prepare the stock solution. pH adjusted to 7 with NaOH and filter sterilized
Propionate	GAM-PROPI	2 g/l	Acid form used to prepare the stock solution. pH adjusted to 7 with NaOH and filter sterilized
Isobutyrate	GAM-ISBUT	1 g/l	Acid form used to prepare the stock solution. pH adjusted to 7 with NaOH and filter sterilized
Isovalerate	GAM-ISVAL	1 g/l	Acid form used to prepare the stock solution. pH adjusted to 7 with NaOH and filter sterilized
Lactate	GAM-LACT	5 g/l	Acid form used to prepare the stock solution. pH adjusted to 7 with NaOH and filter sterilized
Formate	GAM-FORMT	2 g/l	Acid form used to prepare the stock solution. pH adjusted to 7 with NaOH and filter sterilized

Glutamate	GAM-GLUTA	5 g/l	Acid form used to prepare the stock solution. pH adjusted to 7 with NaOH and filter sterilized
**Bile acids**			total concentration in the small intestine after meal between 1 and 10 mM, ie 0.4 to 4 g/L
TCA	GAM-TCA	2 g/l	Primary BA - FXR antagonist. Filter sterilized
GCA	GAM-GCA	2 g/l	Primary BA - FXR antagonist. Filter sterilized
CA	GAM-CA	2 g/l	Primary conjugated BA. Filter sterilized
CDCA	GAM-CDCA	2 g/l	Primary conjugated BA. Filter sterilized
DCA	GAM-DCA	2 g/l	Secondary BA - FXR antagonist. Filter sterilized
LCA	GAM-LCA	2 g/l	Secondary BA - FXR antagonist. Filter sterilized
Taurine	GAM-TAURN	50 mM (6.25 g/l)	Precursor of BA metabolism. Filter sterilized
**Sugars and DF**			
Inulin	GAM-INUL	10 g/l	
Pectin	GAM-PECT	10 g/l	
Mucin	GAM-MUCIN	10 g/l	
Xanthan	GAM-XANTA	10 g/l	
**Compounds of interest**			
Capsaicin	GAM-CAPSI	16 mg/L	Filter sterilized
Urea	GAM-UREA	50 g/l	Filter sterilized
fluoride	GAM-FLUO	2 mg/L	Filter sterilized

Oxalate	GAM-OXAL	5 g/l	Pure oxalic acid is used to make the stock solution. pH of the stock solution is then adjusted to 7 with NaOH. Filter sterilized
L-Tryptophan	GAM-TRPTO	5 g/l	Filter sterilized
L-Tyrosine	GAM-TYRO	5 g/l	Filter sterilized
L-Histidine	GAM-HISTI	5 g/l	Filter sterilized
Caffeine	GAM-CAFFE	10 g/l	Filter sterilized
**pH**			
4	GAM-pH4	NA	GAM-STD plates with pH adjusted to 4 before pouring.
5	GAM-pH5	NA	GAM-STD plates with pH adjusted to 5 before pouring.
6	GAM-pH6	NA	GAM-STD plates with pH adjusted to 6 before pouring.
8	GAM-pH8	NA	GAM-STD plates with pH adjusted to 8 before pouring.
**Other stress**			
Ethanol	GAM-EtInc	NA	Ethanol treated inoculum according to Browne, 2016. The plate contains 1g/l of GCA to stimulate germination of spore formers
Oxygen	GAM-O2	NA	GAM-STD plates incubated aerobically. Selection for facultative anaerobes and strict aerobes.
Nanoparticles	GAM-NANOP	5 mg/l	Spherical 50 nm gold nanoparticles in PBS were used.
Salt high	GAM-NaCl	50 g/l	Plates contains a higher concentration of NaCl. Non halophiles grow at NaCl concentrations >0.2 M, moderate halophiles from 0.2 to 3.5 M NaCl

Low Growth temperature	GAM-30°C	NA	GAM-STD plates incubated anaerobically at 30°C
High Growth temperature	GAM-42°C	NA	GAM-STD plates incubated anaerobically at 42°C
Low nutrient	GAM-10X	NA	GAM-STD diluted 10X to achieve lower nutrient concentration

**Enrichment**			
De Man, Rogosa and Sharpe medium	MRS-STD	NA	Commercial medium for the enrichment of *Bifidobacteria* and *Lactobacilli*.
Bacteroides Bile Esculin	BBE-STD	NA	Commercial medium for the enrichment of Bacteroides fragilis group from
['Erythromycin', 'CA']	ISO-0001	NA	
['Tetracyclin','Ciprofloxacin']	ISO-0002	NA	
['Tetracyclin','Clindamycin']	ISO-0003	NA	
['Ciprofloxacin', 'Clindamycin']	ISO-0004	NA	
['Erythromycin', 'Clindamycin']	ISO-0005	NA	
['Tetracyclin','Ciprofloxacin','Clindamycin']	ISO-0006	NA	
['Erythromycin', 'CA','Ciprofloxacin']	ISO-0007	NA	
['Acetate','Pectin','pH6','10X']	ISO-0008	NA	

['Ciprofloxacin','Pectin','pH6','10X','Fluoride']	ISO-0009	NA	
['Ciprofloxacin','Mucin','pH6','Isovalerate']	ISO-0010	NA	
['Ciprofloxacin','Mucin','pH6','Isovalerate','TCA']	ISO-0011	NA	
['Oxalate','Mucin','pH5','10X']	ISO-0012	NA	
['Ciprofloxacin','Pectin','Inulin','10X']	ISO-0013	NA	
['Ciprofloxacin','Pectin','Inulin','10X','GCA','30C']	ISO-0014	NA	
['Ciprofloxacin','Pectin','Gentamycin','Formate','LCA','42C']	ISO-0015	NA	
['Ciprofloxacin','Xanthan','pH5','Acetate','CDCA']	ISO-0016	NA	
['Ciprofloxacin','Clindamycin','Xanthan','pH5','Acetate','CDCA']	ISO-0017	NA	
['Ciprofloxacin','Gentamycin','Xanthan','pH5','Acetate']	ISO-0018	NA	
['Lactate','Mucin','Fluoride','Na	ISO-0019	NA	

noparticle','10X']			
['Pectin','Inulin','10X','Lactate','TCA']	ISO-0020	NA	
['Clindamycin','Inulin','Tryptophan','Isovalerate','CDCA','42C']	ISO-0021	NA	
['Clindamycin','Inulin','Tryptophan','Oxalate','GCA','42C']	ISO-0022	NA	
['Histidine','TCA']	ISO-0023	NA	
['Ciprofloxacin','Histidine','Sulfamethoxazole','TCA']	ISO-0024	NA	
['Xanthan','10X','30C','CDCA']	ISO-0025	NA	
['Ciprofloxacin','Clindamycin','Inulin','Tryptophan']	ISO-0026	NA	
['Erythromycin','Tryptophan','Mucin','DCA','Isovalerate']	ISO-0027	NA	
['Clindamycin','Tryptophan','Sulfamethoxazole','CDCA']	ISO-0028	NA	
['Ciprofloxacin','Gentamycin','Inulin','Tryptophan']	ISO-0029	NA	

['Clindamycin','Inulin','Sodium salt','CDCA']	ISO-0030	NA	
['Pectin','Inulin','pH5','10X','30C','TCA']	ISO-0031	NA	
['Tetracyclin','Clindamycin','Mucin','pH5']	ISO-0032	NA	
