# Supplementary material for: Metagenome-guided culturomics for the targeted enrichment of gut microbes
Source: Nat Commun. 2025 Jan 14;16:663. doi: 10.1038/s41467-024-55668-y (PMC11733127; doi:10.1038/s41467-024-55668-y)
Supplement: Supplementary file 2 — Reporting Summary [file 41467_2024_55668_MOESM2_ESM.pdf]

nature portfolio

Corresponding author(s): Morten Otto Alexander Sommer

Last updated by author(s): Dec 18, 2024

Reporting Summary

Nature Portfolio wishes to improve the reproducibility of the work that we publish. This form provides structure for consistency and transparency in reporting. For further information on Nature Portfolio policies, see our Editorial Policies and the Editorial Policy Checklist.

Statistics

For all statistical analyses, confirm that the following items are present in the figure legend, table legend, main text, or Methods section.

n/a	Confirmed	
☐	☒	The exact sample size (n) for each experimental group/condition, given as a discrete number and unit of measurement
☐	☒	A statement on whether measurements were taken from distinct samples or whether the same sample was measured repeatedly
☐	☒	The statistical test(s) used AND whether they are one- or two-sided<br>*Only common tests should be described solely by name; describe more complex techniques in the Methods section.*
☐	☒	A description of all covariates tested
☐	☒	A description of any assumptions or corrections, such as tests of normality and adjustment for multiple comparisons
☐	☒	A full description of the statistical parameters including central tendency (e.g. means) or other basic estimates (e.g. regression coefficient) AND variation (e.g. standard deviation) or associated estimates of uncertainty (e.g. confidence intervals)
☐	☒	For null hypothesis testing, the test statistic (e.g. F, t, r) with confidence intervals, effect sizes, degrees of freedom and P value noted<br>*Give P values as exact values whenever suitable.*
☒	☐	For Bayesian analysis, information on the choice of priors and Markov chain Monte Carlo settings
☒	☐	For hierarchical and complex designs, identification of the appropriate level for tests and full reporting of outcomes
☐	☒	Estimates of effect sizes (e.g. Cohen's d, Pearson's r), indicating how they were calculated

Our web collection on statistics for biologists contains articles on many of the points above.

Software and code

Policy information about availability of computer code

Data collection	Data tracking of all samples from sampling to sequencing analysis was handled via barcoding and recorded in Benchling. No statistical or randomization methods were used to design the experiments.
Data analysis	No rarefaction of reads was performed. Sequencing data were processed using the NG-Metaprofiler to obtain taxonomc profiles. Data handling, calculations and statistical modeling were performed using Python 3.8 and the packages Numpy 1.22, Pandas 1.3, Scipy 1.7 and Scikit-learn 1.0. Ecological metrics such as alpha diversity, beta diversity, phylogenetic tree, PERMDISP, PERMANOVA and ANCOM were calculated using Scikit-bio 0.5. Phylogenetic tree was visualized with iTOL59. All plots were generated with the python packages Matplotlib 3.5, Seaborn 0.11 and Plotly 5.5. PCAs were generated using Scikit-learn. Figures were made using Adobe Illustrator CC. The investigators were not blinded to sample allocation during the experiments or the analysis.

For manuscripts utilizing custom algorithms or software that are central to the research but not yet described in published literature, software must be made available to editors and reviewers. We strongly encourage code deposition in a community repository (e.g. GitHub). See the Nature Portfolio guidelines for submitting code & software for further information.

Data

Policy information about availability of data

All manuscripts must include a data availability statement. This statement should provide the following information, where applicable:

- Accession codes, unique identifiers, or web links for publicly available datasets
- A description of any restrictions on data availability
- For clinical datasets or third party data, please ensure that the statement adheres to our policy

The sequencing data generated in this study has been deposited in SRA database under the BioProject accession number: PRJNA1077691 [https://www.ncbi.nlm.nih.gov/bioproject/PRJNA1077691/]. Source data are provided with this paper.

Research involving human participants, their data, or biological material

Policy information about studies with human participants or human data. See also policy information about sex, gender (identity/presentation), and sexual orientation and race, ethnicity and racism.

Reporting on sex and gender	Sample donations were performed by individuals matching the following set of criteria, with 4 males and 2 females: Participants were selected to be considered healthy individual and therefore to fulfill the following self-declared inclusion criteria prior to donation: males or females, aged 20-60, not following a particular diet regimen and without underlying or chronic condition, not having undergone antibiotic, antiviral, antifungal or antiparasitic treatment over the 12 months prior donation, not suffering of gastrointestinal pain, discomfort, intolerance or been subject to a surgical procedure.
Reporting on race, ethnicity, or other socially relevant groupings	As per university and EU regulation, personal data on participants regarding race, ethnicity or socially relevant groupings were not used for the analysis.
Population characteristics	Anonymous participants were selected to be considered healthy individual and therefore to fulfill the following self-declared inclusion criteria prior to donation: males or female and aged 20-60
Recruitment	Participants self-reported to not follow a particular diet regimen and had no self-reported underlying or chronic condition, not having undergone antibiotic, antiviral, antifungal or antiparasitic treatment over the 12 months prior donation, not suffering of gastrointestinal pain, discomfort, intolerance or been subject to a surgical procedure.
Ethics oversight	The study was pre-assessed by De Videnskabsetiske Komiteer for Region Hovedstaden under case reference number 22013134. The committee concluded that the project was not a health science research project and therefore did not require ethical approval.<br><br>The study adhered to the ethical guidelines of the Danish Technical University and complied with all applicable EU regulations. Data collection and processing were conducted in full accordance with the GDPR. Written informed consent was obtained from all participants, explicitly permitting the use of their samples for research purposes. Participation was entirely voluntary, with no financial or other forms of compensation provided. Sample were anonymized, this decision aligns with the committee's guidelines, which state that research involving only completely anonymous human biological material collected according to relevant legislation does not require reporting.

Note that full information on the approval of the study protocol must also be provided in the manuscript.

Field-specific reporting

Please select the one below that is the best fit for your research. If you are not sure, read the appropriate sections before making your selection.

[x] Life sciences   [ ] Behavioural & social sciences   [ ] Ecological, evolutionary & environmental sciences

For a reference copy of the document with all sections, see nature.com/documents/nr-reporting-summary-flat.pdf

Life sciences study design

All studies must disclose on these points even when the disclosure is negative.

Sample size	No statistical or randomization methods were used to design the experiments. No sample size calculations were performed to determine how many donors should be included. We assessed that it would be feasible to perform one run of Novaseq Illumina Sequencing for each round of the study. As we aimed at having deep sequencing coverage, we determined that stool samples would be appropriate, considering that we have 50 culture conditions for each donor. Microbial communities from stool samples of six healthy human donors were collected. Participants were requested to self-sample using a kit composed of a container containing antioxidant solution, a GasPak anaerobic pouch and nitrile gloves. Once the donation was completed, participants anonymously deposited the sample in a dedicated box stored at 4°C for subsequent processing over the next hour the same day to preserve sample viability. The samples were placed in anaerobic conditions in a Don Whitley A95 anaerobic chamber at ambient temperature (5% Hydrogen, 20% Carbon dioxide, 75% Nitrogen). The samples were then prepared for cryopreservation at -80°C.
Data exclusions	Anonymous participants should have not been following a particular diet regimen and without underlying or chronic condition, not having undergone antibiotic, antiviral, antifungal or antiparasitic treatment over the 12 months prior donation, not suffering of gastrointestinal pain, discomfort, intolerance or been subject to a surgical procedure.
Replication	The experiment was conducted in 6 different donors in order to account for inter-personal variability of gut microbiota between donors. Since the study was designed to assess variability across different individuals, the concept of replication doesn't directly apply in this context.<br><br>Covariates were not controlled, as they were not relevant to the study's objectives. In compliance with university and EU regulations, personal data from participants were excluded from the analysis to ensure privacy.
Randomization	Participants anonymously deposited the sample in a dedicated box stored at 4°C for subsequent processing. No statistical or randomization methods were used to design the experiments.
Blinding	The investigators were not blinded to sample allocation during the experiments or the analysis.

Reporting for specific materials, systems and methods

We require information from authors about some types of materials, experimental systems and methods used in many studies. Here, indicate whether each material, system or method listed is relevant to your study. If you are not sure if a list item applies to your research, read the appropriate section before selecting a response.

Materials & experimental systems

n/a	Involved in the study
☒	☐ Antibodies
☒	☐ Eukaryotic cell lines
☒	☐ Palaeontology and archaeology
☒	☐ Animals and other organisms
☒	☐ Clinical data
☒	☐ Dual use research of concern
☒	☐ Plants

Methods

n/a	Involved in the study
☒	☐ ChIP-seq
☒	☐ Flow cytometry
☒	☐ MRI-based neuroimaging

Plants

Seed stocks	N/A
Novel plant genotypes	N/A
Authentication	N/A
